# Effects of forest bathing (Shinrin-yoku) in stressed people

**DOI:** 10.3389/fpsyg.2024.1458418

**Published:** 2024-11-01

**Authors:** Luca Queirolo, Teresa Fazia, Andrea Roccon, Elisa Pistollato, Luigi Gatti, Luisa Bernardinelli, Gastone Zanette, Franco Berrino

**Affiliations:** ^1^Department of Neurosciences, University of Padua, Padua, Italy; ^2^Department of Philosophy, Sociology, Education and Applied Psychology, University of Padua, Padua, Italy; ^3^Department of Brain and Behavioral Sciences, University of Pavia, Pavia, Italy; ^4^Department of Medicine, University of Padua, Padua, Italy; ^5^Associazione La Grande Via, Milan, Italy

**Keywords:** stress, nature, forest, quality of life, EDA, HRV, Shinrin-yoku

## Abstract

**Aim:**

This study aims to explore the physiological effect of forest bathing on stress management.

**Methods:**

A total of 29 volunteers participated in this pre-post design, which lacked a control group. Several physiological parameters were recorded, including heart rate (HR), heart rate variability (HRV), electrodermal activity (EDA), blood pressure (BP), immunoglobulin A (IGA), and salivary cortisol (sCort). Additionally, the Perceived Stress Scale (PSS-10) was administered before forest exposure. Measurements were taken before and after participants spent 2 days fully immersed in a forest environment. To further assess stress management, participants completed a Mental Arithmetic Task (MAT) before and after forest immersion, during which EDA, HRV, and HR were monitored using an Empatica E4 wristband. Measurements were taken at baseline, during MAT, and afterward (recovery).

**Results:**

Participants exhibited moderate perceived stress levels before forest immersion (mean PSS-10 = 21.22, SD = 3.78). Post-forest exposure, there was a significant decrease in sCort (*p* < 0.05) and EDA (*p* < 0.001), while HRV increased (*p* < 0.001), and diastolic blood pressure rose (*p* < 0.05). ANOVA results from the MAT showed a significant increase in parasympathetic activity across all conditions post-immersion (*p* < 0.05), except during recovery, while EDA decreased in all conditions post-forest exposure (*p* < 0.05).

**Conclusion:**

Shinrin-yoku significantly improved stress management at a physiological level and could be a valuable intervention for individuals experiencing stress. However, longitudinal studies with a control group are necessary to determine whether these effects are sustained over time. Nonetheless, this study highlights the potential benefits of forest immersion for stress reduction by enhancing sympathovagal balance and the adaptability of the stress response system.

## Graphical abstract

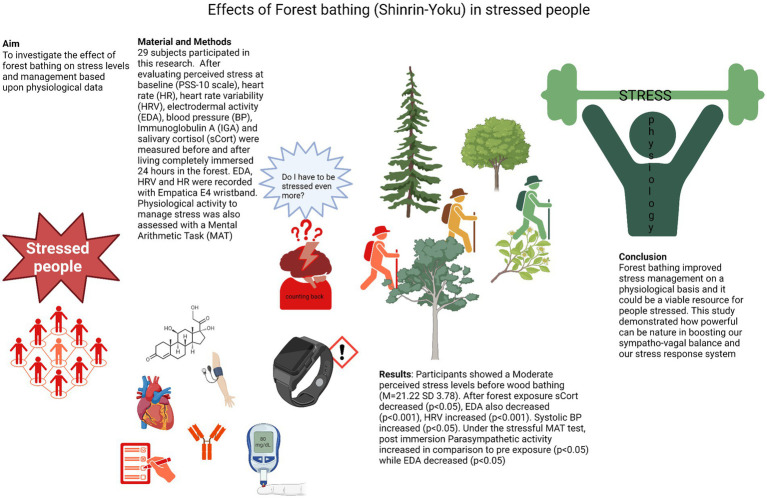

Created with biorender.com.

## Introduction

Contact with nature appears to have beneficial effects on the body 1 (Largo-Wight et al., 2011; Bakir-Demir et al., 2021; de la Osa et al., 2024), enhancing the quality of life, physical and mental health, and overall wellbeing (White et al., 2023; Li et al., 2021). Viewing natural environments may induce changes in the autonomic nervous system through vagal modulation (Gladwell et al., 2012). Lygum et al. (2023) also highlighted the benefits of outdoor work for office workers, including increased creativity and cooperation with colleagues. Indeed, factors such as social integration, better air quality, reduced stress, and physical activity likely contribute to the positive effects of natural contact (Kuo, 2015). In one study, female workers experiencing stress reported improved mental wellbeing after forest bathing, although no changes in HRV were observed, and cortisol levels increased, possibly due to other unexamined factors (Jung et al., 2015).

Kolster et al. (2023) found that nature-based interventions can enhance the mental wellbeing of patients with poor mental health. Living in contact with nature has also been shown to protect against hypertension, especially in older adults (Bauwelinck et al., 2020). Lee et al. (2011) found reductions in cortisol levels and increases in HRV, indicating decreased sympathetic activity in individuals engaging in forest bathing. Several authors have broadly discussed the positive psychophysiological effects of forest bathing (Lee et al., 2011; Park et al., 2010).

Despite extensive evidence of improved wellbeing, there is a lack of studies that monitor the adaptation of the stress response system using a validated stress-inducing procedure (Williams et al., 2004; Fuller, 1977) and portable devices before and after forest bathing. However, despite some methodological discrepancies, reviews and meta-analyses consistently support the strong effect of forest bathing on parasympathetic (Farrow and Washburn, 2019) nervous system activity and a significant reduction in the stress response system (Antonelli et al., 2019), particularly in cortisol levels.

Psychophysiology, an interdisciplinary field linking psychological aspects with patterns of physiological regulation, and vice versa, is promising for explaining how dysfunctional physiological activation can negatively affect health. It also predicts poorer adaptation to stressors, ultimately leading to health risks (Rosenman et al., 1966; Lyness, 1993; Molinari et al., 2007). The definition of stress, as derived from Selye’s studies, describes it as an individual psychophysiological response mediated by the autonomic nervous system and the endocrine system in reaction to environmental demands that threaten homeostasis (Selye, 1957).

A better autonomic balance, mediated by improved cognitive flexibility and related brain activity, is associated with improved stress management, emotional regulation, attention skills, and overall mental health and wellbeing (Thayer et al., 2009; Thayer et al., 2012). Specifically, images of natural environments and walking in nature reserves improve attention, blood pressure balance, and emotional control. A more in-depth study of the relationship between forest bathing and the stress response system could enhance our understanding of how healthier lifestyles contribute to improved self-regulation and reduced mortality. Forest bathing has been proven to be beneficial for cardiovascular and metabolic health (Li et al., 2016).

Our project’s objective is to evaluate how psychological variables and physiological correlates highlight the impact of nature on stress adaptation responses. We will achieve this by evaluating various indices identified in the literature as stress biomarkers across different situations and environments (Queirolo et al., 2023; Hanshans et al., 2024; De Looff et al., 2018; Bhoja et al., 2020; Klimek et al., 2023; Queirolo et al., 2024). Although there is evidence of nature’s positive effects, the mechanisms and pathways still need to be fully understood. Therefore, this research aims to elucidate the effect of forest bathing on the stress response system and how the autonomic nervous system is modulated by forest bathing.

## Materials and methods

Psychological data and physiological measures were collected from 29 healthy participants (10 men and 19 women, mean age = 48.8, SD = 9) who attended the event “La Via delle Foreste” from August 14 to 15, 2023. The study was approved by the Ethics Committee of the Department of Brain and Behavioral Sciences at the University of Pavia (Approval No. 130/23).

***The exclusion criteria*** were as follows:

- Current mental disorders (determined through an interview with a psychologist)
- Heart disease, which could affect the stress response
- Addictions to psychotropic substances or use of rhythm modulators
- History of organ transplant.

### Methodology

At baseline (pre-forest bathing), participants were evaluated at the Mausolea, a building located near the Casentino forest in Tuscany, to assess their psychophysiological state. This evaluation included the Perceived Stress Scale (Cohen et al., 1994) (PSS-10), salivary measurement of immunoglobulin A (Ng et al., 1999; Chojnowska et al., 2021), cortisol (collected while fasting at 9 a.m.), as well as glycemia and blood pressure. The PSS-10 scale categorizes (Mondo et al., 2021) stress levels as follows: scores from 0 to 13 indicate low stress, 14–25 indicate moderate stress and 26 or above indicate high perceived stress.

Participants also performed the MAT stress test (Williams et al., 2004; Fuller, 1977), which involves counting backward by subtracting 13 from 1,007 and restarting after a mistake while wearing an Empatica E4 wearable bracelet. Empatica E4 device recorded physiological parameters following this procedure: baseline (5 min), stress stimulation (5 min, MAT test), and recovery (5 min).

After the baseline evaluations, the participants spent 1 day and one night in nature under the supervision of one of us (FB) within the La Grande Via project. They engaged their senses mindfully while walking for 3 h in the Casentino forest, rich in white firs and beach trees. They experienced moments of rest, contemplation, yoga practices, physical tree contact, meditation, and mantra chanting. They followed a diet combining Mediterranean and macrobiotic diets and slept one night in nature (in a hammock). Back at the Mausolea the following morning, participants underwent the same evaluation as at baseline, except for the PSS-10. Salivary cortisol was collected again at 9 o’clock while fasting.

### Physiological measures

Physiological parameters were derived using Empatica E4, a smartwatch wearable device that measures electrodermal activity (EDA), blood pulse volume [from which heart rate (HR) and heart rate variability (HRV) are derived], temperature, and movement (Bhoja et al., 2020). EDA is a property of the skin that indicates variations in electrical conduction in response to sweat secretions and is a pure sympathetic index (Klimek et al., 2023; Boucsein, 2012; Shields et al., 1987). HRV represents the variation in the time interval between heartbeats, a parasympathetic index reflecting vagal activity, and is measured by the root mean square of successive differences between beat intervals (Acharya et al., 2006; Umetani et al., 1998; Ernst, 2017). Participants wore the monitoring device on their non-dominant hands before and after forest bathing while performing the stressful MAT test.

Measurements were divided into three conditions: “baseline,” “N-back task” (the counting task from the MAT test), and “recovery.” HR was expressed in beats per minute (bpm) and derived using Empatica algorithms from the blood volume pulse. HRV was calculated as the root mean square of successive differences between normal heartbeats (RMSSD) by calculating each successive time difference between heartbeats in milliseconds (ms) from the inter-beat interval (IBI) over a short-term period of 30 s.

The PPG sensor used to detect blood volume pulse is known to be subject to missing data due to movement or pressure artifacts (Chojnowska et al., 2021). Artifacts were removed by discarding zero values and other single-point data outliers. The analysis of EDA involved extracting the skin conductance level. The electrodes used were silver coated with copper underlay on brass. The threshold for the amplitude of a significant signal was set to a minimum rise of 0.005 μSiemens. EDA values were then normalized using the min-max method. Physiological data were down-sampled to 1 Hz and labeled as belonging to the “baseline,” “N-back task,” or “recovery” condition. Data from each subject were then aggregated into periods and groups for analysis.

### Statistical analysis

First, the assumed normality of the recorded variables was tested using the Shapiro–Wilk test. Normally distributed variables were analyzed using a Student T-test, while non-normally distributed variables were analyzed with non-parametric tests (Wilcoxon, Welch-t test, Mann–Whitney). The normality of residuals and homoscedasticity of variance were checked before fitting a one-way ANOVA model. A repeated measures ANOVA (pre- and post-forest experience) with condition levels (“baseline,” “N-back,” “recovery”) was used to determine whether the primary outcomes (dependent variables: physiological variables; EDA, HRV, and systolic BP measured during the MAT stress test) significantly differed across the three conditions. The correlation between self-reported questionnaire scores and physiological variables was estimated using Pearson’s r.

## Results

**Figure 1:**
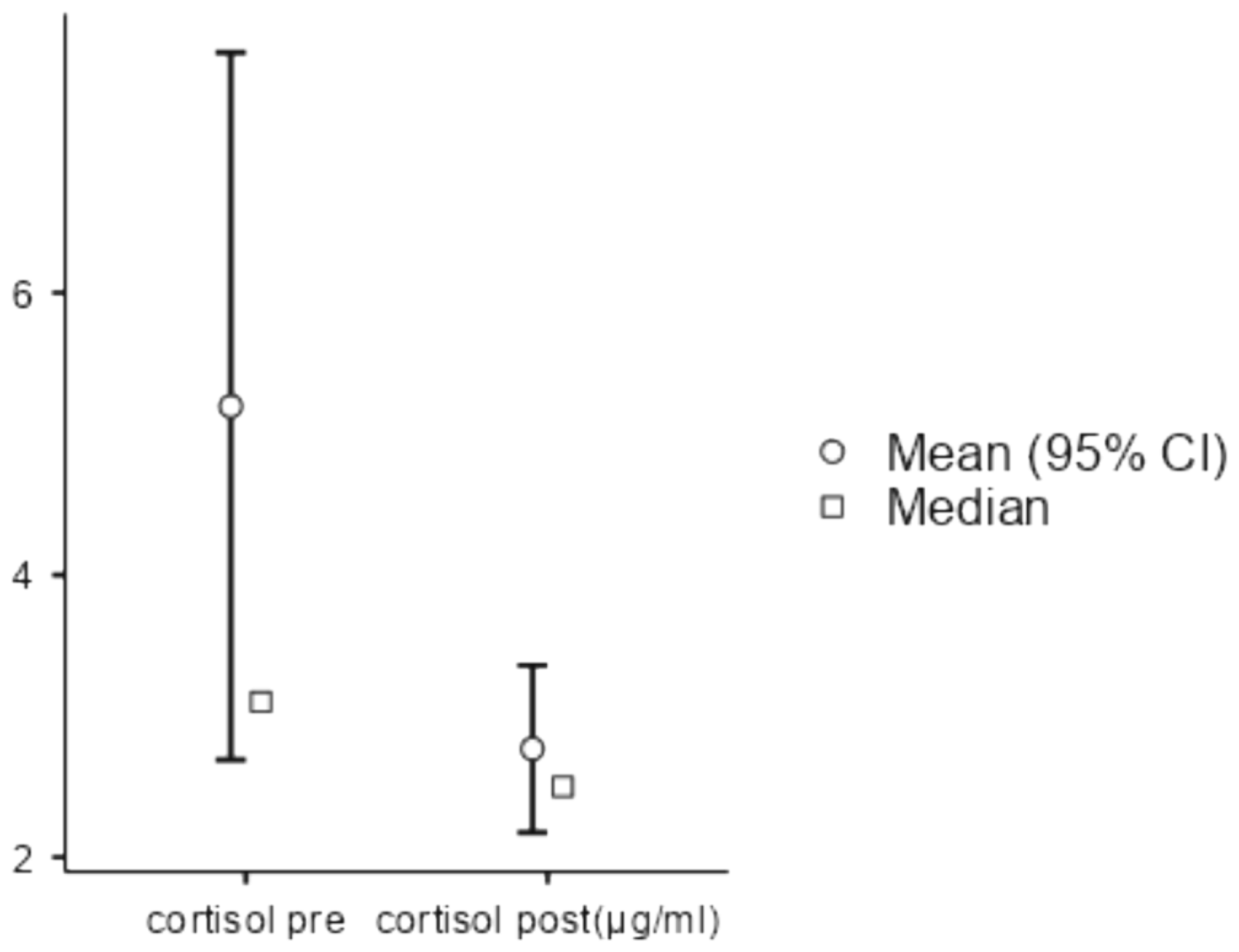
Salivary cortisol comparison between pre- and post-nature exposure. After checking normality assumptions with the Shapiro–Wilk test, the Wilcoxon rank test was conducted, statistics 237, *p* < 0.05, DF = 24. Cortisol levels decreased from 5.2 μg/dL to 2.77 μg/dL.

Differences in fasting glycemia, immunoglobulin A (IGA), and systolic blood pressure before and after forest bathing were not statistically significant. Regarding cortisol, four out of 58 samples were damaged during post-forest exposure data collection, resulting in missing data for those participants. However, Scort significantly decreased (*W* = 237, *p* < 0.05, DF = 24) when comparing pre- and post-forest bathing levels, indicating a reduction in stress hormone activity (see Figure 1).

**Figure 2:**
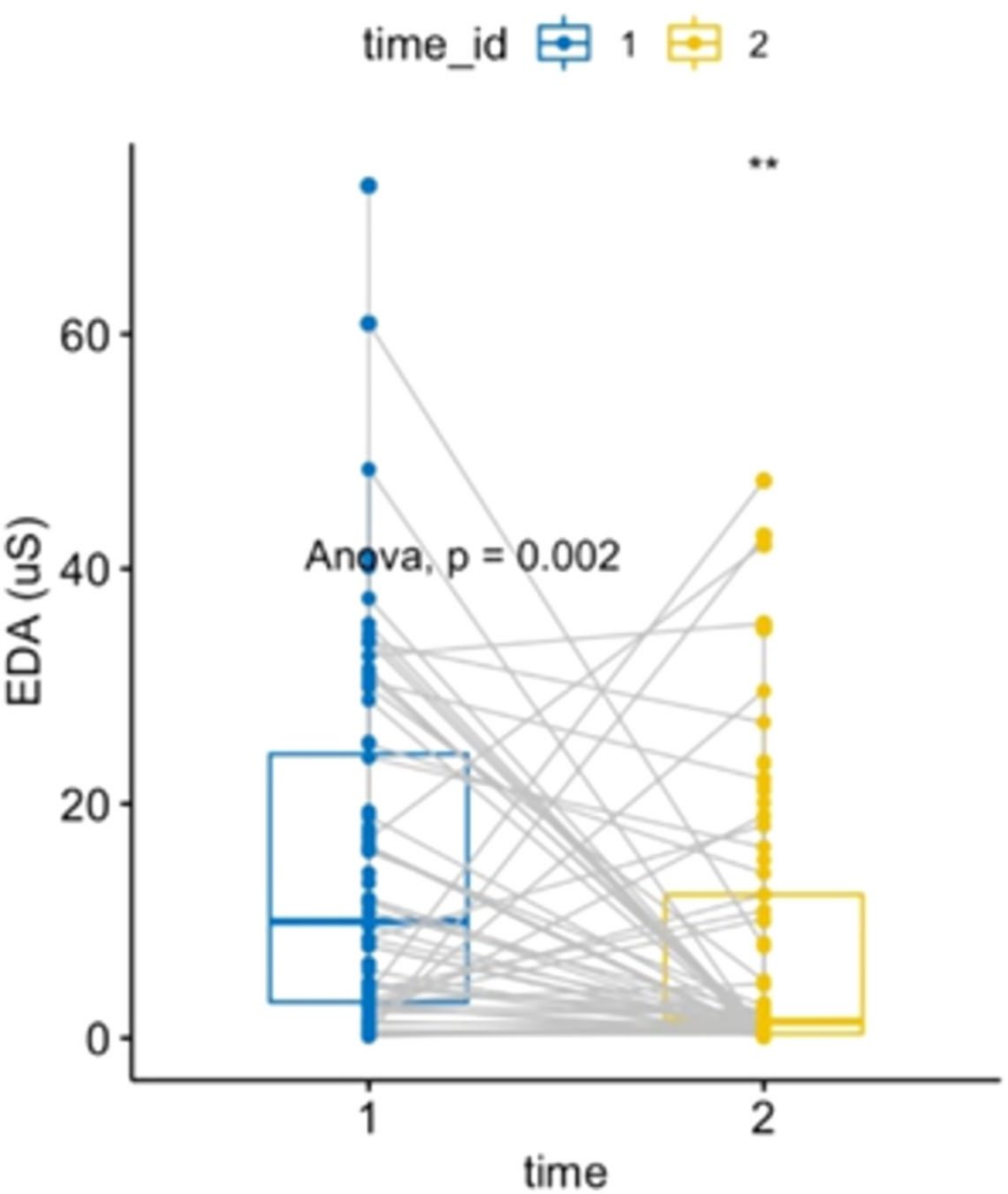
Electrodermal activity: two-way ANOVA, repeated measures within subjects: time [2 levels (1 = pre-nature exposure, 2 = post-nature exposure), condition (1 level)], *p* = 0.0017284, *F*-value = 10.196, DF = 1. Electrodermal activity comparison between pre- and post-exposure showed a decrease of 6.96 μSiemens (IC 95%: −11.262, −2.65).

A one-way ANOVA of EDA comparing pre- and post-forest bathing was performed after confirming the data’s normality, residual, and variance homoscedasticity. EDA activity decreased (*p* = 0.002, *F*-value = 10.196, DF = 1), demonstrating the effect of forest bathing on reducing sympathetic activity (see Figure 2).

**Figure 3:**
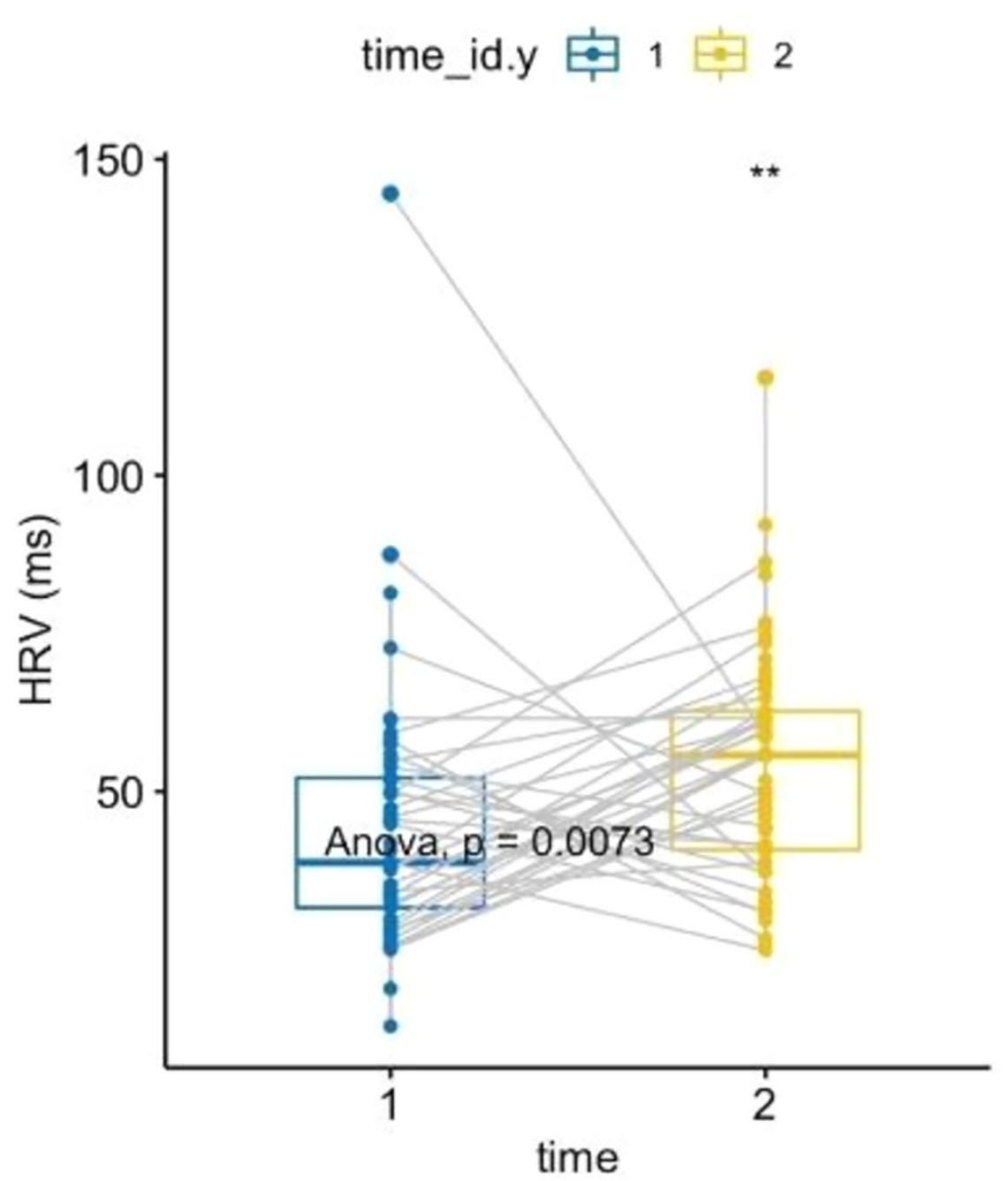
Heart rate variability: two-way ANOVA, repeated measures within subjects: time [2 levels (1 = pre-nature exposure, 2 = post-nature exposure), condition (1 level)], *p* = 0.0073, *F*-value = 7.49, DF = 1. Heart rate variability comparison between pre- and post-exposure showed an increase of 9.722377 ms (IC 95%: 2.682, 16.76).

**Figure 4:**
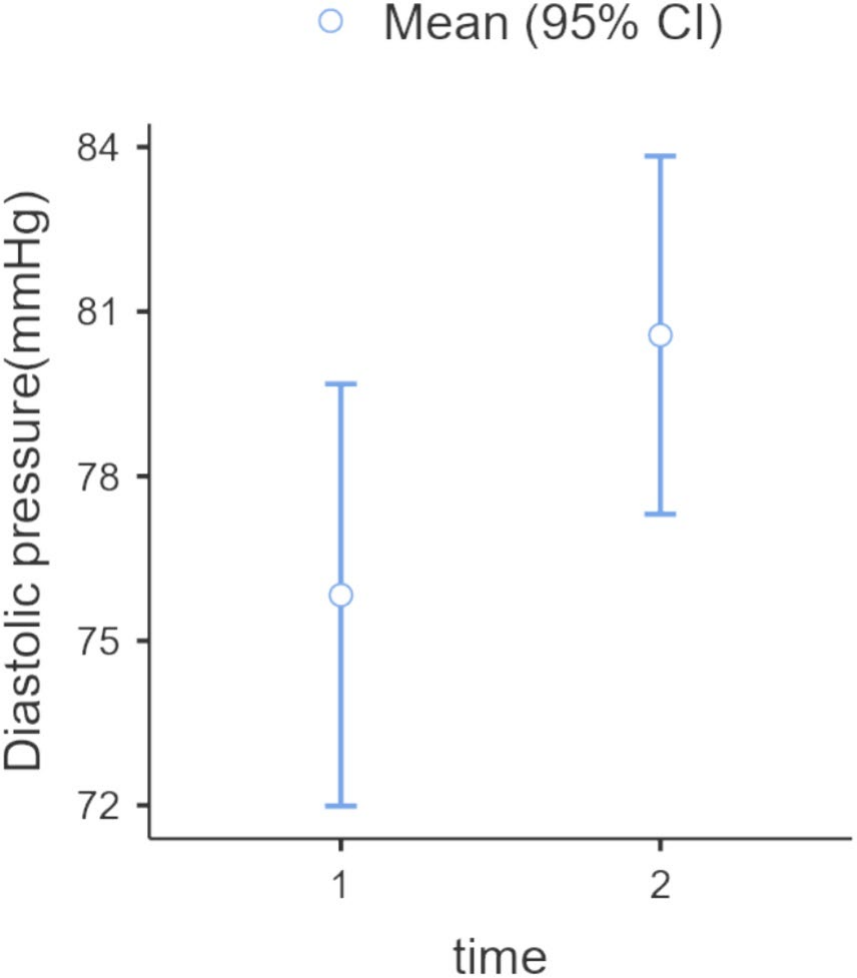
Diastolic blood pressure: one-way ANOVA (Welch), repeated measures, within-subjects: time (2 levels: 1 = pre-nature exposure, 2 = post-nature exposure), *p* = 0.027, *F*-value = 5.14, DF = 56. The difference was 5.55 mmHg (IC 95% 2.58, 8.52).

**Figure 5:**
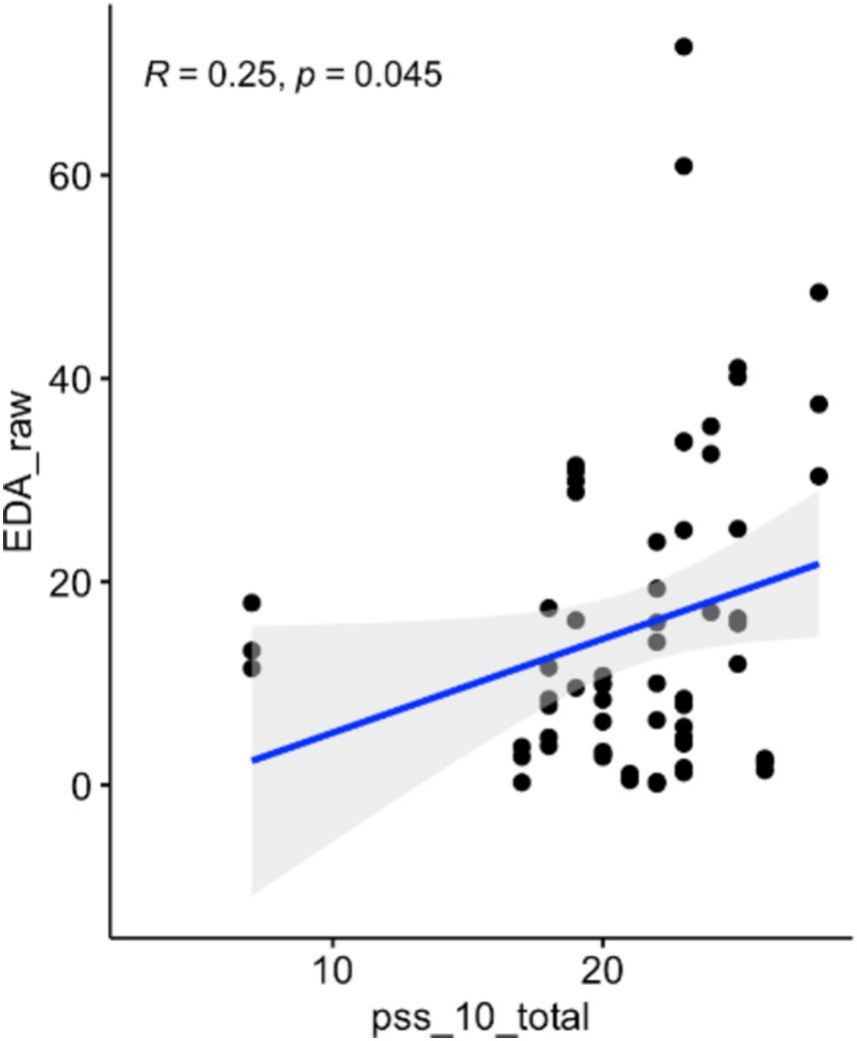
Correlation between electrodermal activity and PSS-10 scale: *R* = 0.25, *p* < 0.05.

A one-way ANOVA of HRV, after verifying all assumptions, showed an increase in parasympathetic activity post-forest bathing (*p* = 0.0073, *F*-value = 7.49, DF = 1), see Figure 3. Similarly, a non-parametric one-way ANOVA of diastolic BP (after confirming all assumptions) indicated an increase (*p* = 0.027, *F*-value = 5.16, DF = 1) (see Figure 4). A correlation between the PSS-10 Scale and EDA was also found (*R* = 0.25, *p* < 0.05), (see Figure 5). EDA and HRV ANOVA models (pre/post-Shinrin-yoku) with condition levels (“baseline,” “N-back,” “recovery”), both showed significant effects of time and condition (*p* < 0.05).

**Figure 6:**
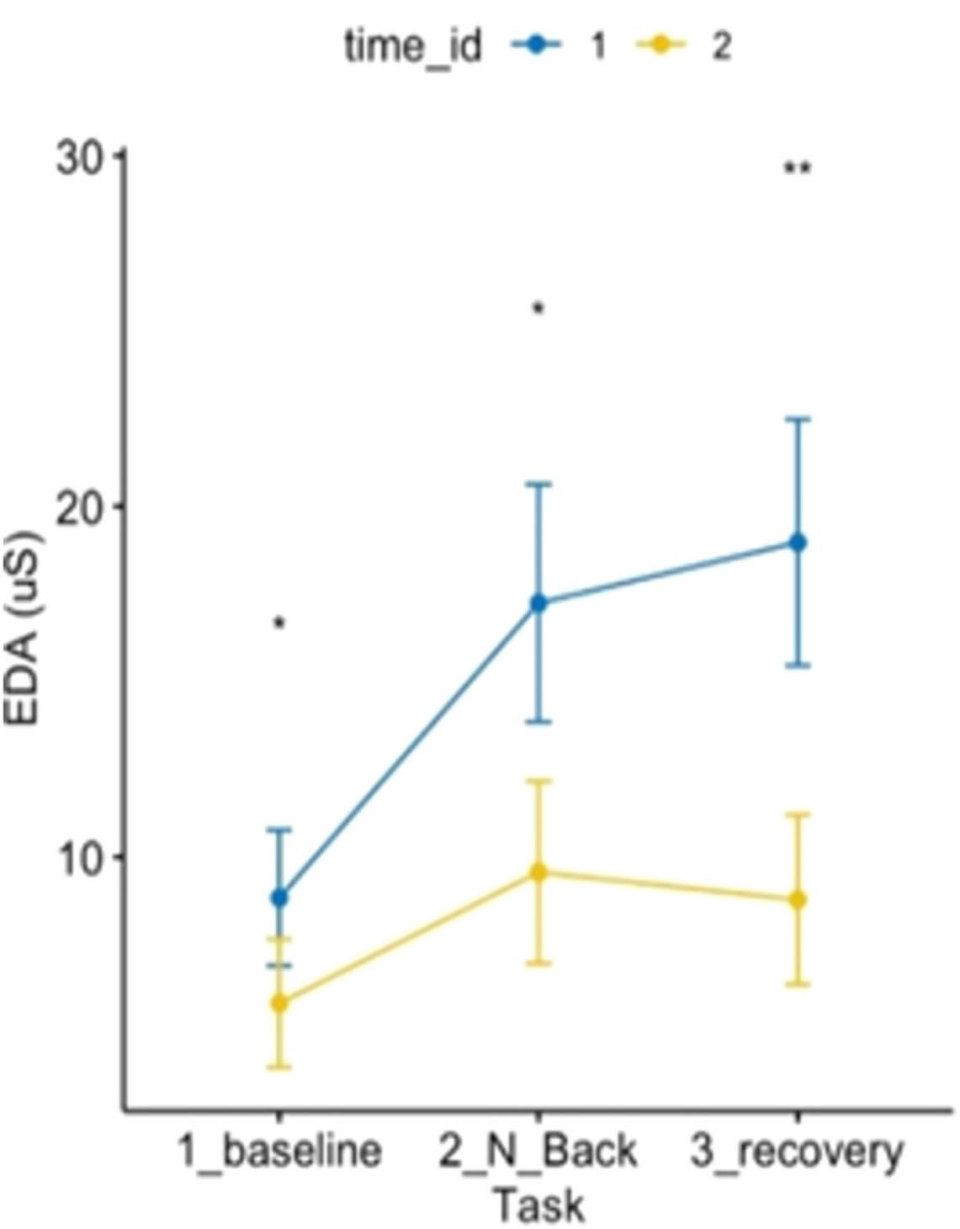
ANOVA 3 × 2 related to electrodermal activity showed an effect of time and condition on electrodermal activity *p* < 0.05.

Specifically, EDA levels (see Figure 6) during baseline, MAT test, and recovery post-forest exposure were lower than those pre-forest bathing. Additionally, the increase in EDA during the stressful MAT test was lower post-exposure than pre-exposure. The recovery phase pattern reversed: while EDA typically increased post-MAT (pre-Shinrin-yoku), it decreased post-forest bathing.

**Figure 7:**
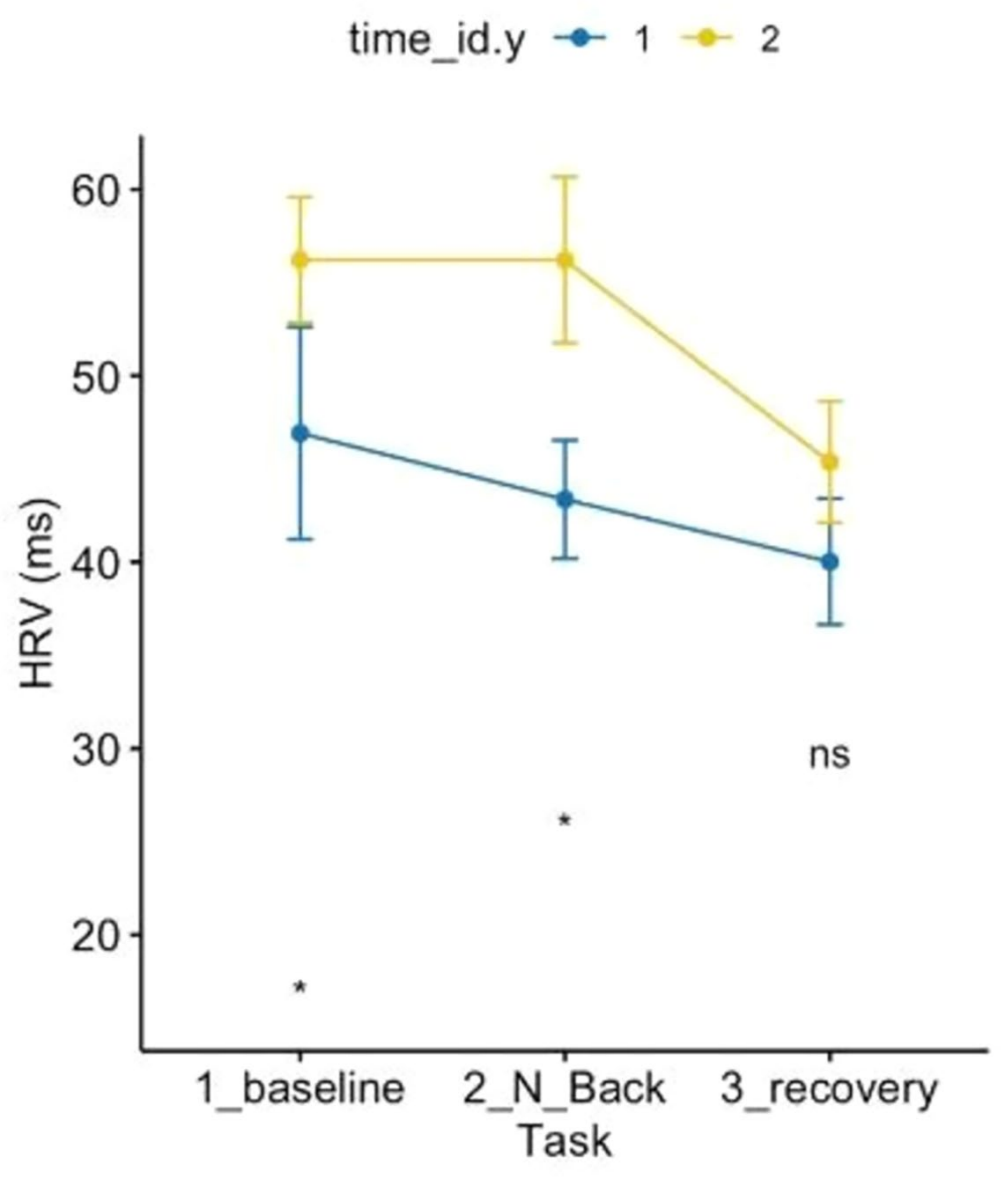
ANOVA 3 × 2 related to heart rate variability showed the effects of time and condition *p* < 0.05.

Regarding the HRV ANOVA model (see Figure 7), a contrasting pattern was observed, except for the recovery phase, which did not differ. Baseline and MAT test conditions pre vs. post-Shinrin-yoku differed significantly with increased parasympathetic activity post-forest bathing compared to pre-forest bathing at baseline and under the MAT test. Furthermore, the pattern over time differed: while HRV would have decreased from baseline to MAT test pre-Shinrin-yoku (indicating stress, shown by increased sympathetic activity), post-forest bathing HRV maintained baseline values, showing a different pattern.

## Discussion

The literature provides several indications that forest bathing is beneficial to human health. Some studies have observed reductions in oxidative stress and pro-inflammatory markers in both healthy individuals and patients (Mao et al., 2012; Jia et al., 2016). Other studies have explored whether Shinrin-yoku (forest bathing) could be a viable intervention for reducing burnout among physicians and healthcare professionals. While no significant changes were found in burnout questionnaire scores, participants overwhelmingly reported decreased stress levels and improved mental wellbeing (Kavanaugh et al., 2022). Some researchers have suggested that forest bathing is useful, but there may be a seasonal effect on the relationship between forest bathing and wellbeing (Oomen-Welke et al., 2022). Another study showed positive effects on hypertension (Mao et al., 2012). In our study, we replicated the reduction in cortisol levels but observed an increase in diastolic blood pressure (within the normal range), which contrasts with previous data (Horiuchi et al., 2015).

Several patterns of stress response have been elucidated in the literature (Hartig et al., 2003; Li et al., 2011). Increased electrodermal activity (EDA) has been shown to differentiate performance under workload stress, such as braking ability (Fine et al., 2022). Another study found that increased EDA is inversely correlated with steadiness in anxious individuals (Noteboom et al., 2001). EDA has also been shown to distinguish workload performance in braking tasks (Collet et al., 2014). Furthermore, a decrease in EDA has been linked to improved error-monitoring competency (Holper et al., 2013), while increased EDA has been associated with lower performance in mathematical accuracy (Pizzie and Kraemer, 2021). Overall, EDA appears to be a sensitive marker for evaluating changes in sympathetic system activation under stress (Visnovcova et al., 2016).

Focusing on the parasympathetic nervous system, a decrease in HRV is usually observed under stressful situations (Taelman et al., 2011; Taelman et al., 2009; Shaffer et al., 2014). Numerous studies have emphasized that increased parasympathetic tone is associated with improved performance (Thayer et al., 2012; De Couck et al., 2019; Costa et al., 2021; Schaich et al., 2020; Weber et al., 2010; Beauchaine and Thayer, 2015). In line with our results, we confirm the physiological improvements highlighted by previous authors, as discussed in the introduction and discussion, and the enhanced ability to manage stress. Participants demonstrated improved competence in handling stressors after forest bathing. Under stress, the parasympathetic system did not decrease post-forest exposure compared to pre-exposure. Furthermore, the increase in sympathetic activity was lower after forest bathing than before. This suggests that the system requires less energy to respond to potential threats, reflecting greater modulatory control by the parasympathetic branch of the nervous system and reduced reliance on the sympathetic response. These findings align with the biopsychosocial model of arousal regulation (Blascovich and Tomaka, 1996).

In summary, sympathetic activity after forest bathing was lower, as the body adopted a calmer strategy in the face of stressors, recognizing it had the necessary resources (increased parasympathetic activity) to cope with the stress. Notably, during the recovery phase, parasympathetic activity returned to levels comparable to pre-exposure, likely due to the corresponding decrease in sympathetic activity during post-forest bathing recovery. This pattern contrasts with the pre-forest exposure recovery phase, where sympathetic activity increased while parasympathetic activity decreased, indicating reduced adaptability to cope with stress.

## Conclusion

This study highlights the positive effects of nature on stress regulation at a physiological level. However, it has some limitations that need to be considered, including the absence of a control group, the inability to control for confounders [as suggested by He et al. (2024)], and the lack of a qualitative description of the interactions between multiple interventions (Simonienko et al., 2023). This study evaluated the combined effect of forest bathing, walking, meditation, and diet.

Based on the various indices analyzed and the implementation of the experimental methodology, we can reasonably conclude that forest bathing in Casentino Park reduced stress levels on a hormonal basis, improved the ability to manage stressors, and enhanced sympathovagal balance.

## Data Availability

The raw data supporting the conclusions of this article will be made available by the authors without undue reservation.
